# Supplementary material for: Deciphering the Path of S-nitrosation of Human Thioredoxin: Evidence of an Internal NO Transfer and Implication for the Cellular Responses to NO
Source: Antioxidants (Basel). 2022 Jun 24;11(7):1236. doi: 10.3390/antiox11071236 (PMC9311519; doi:10.3390/antiox11071236)
Supplement: Supplementary file 1 [file antioxidants-11-01236-s001.zip › antioxidants-1709246-supplementary.pdf]

Supporting Information

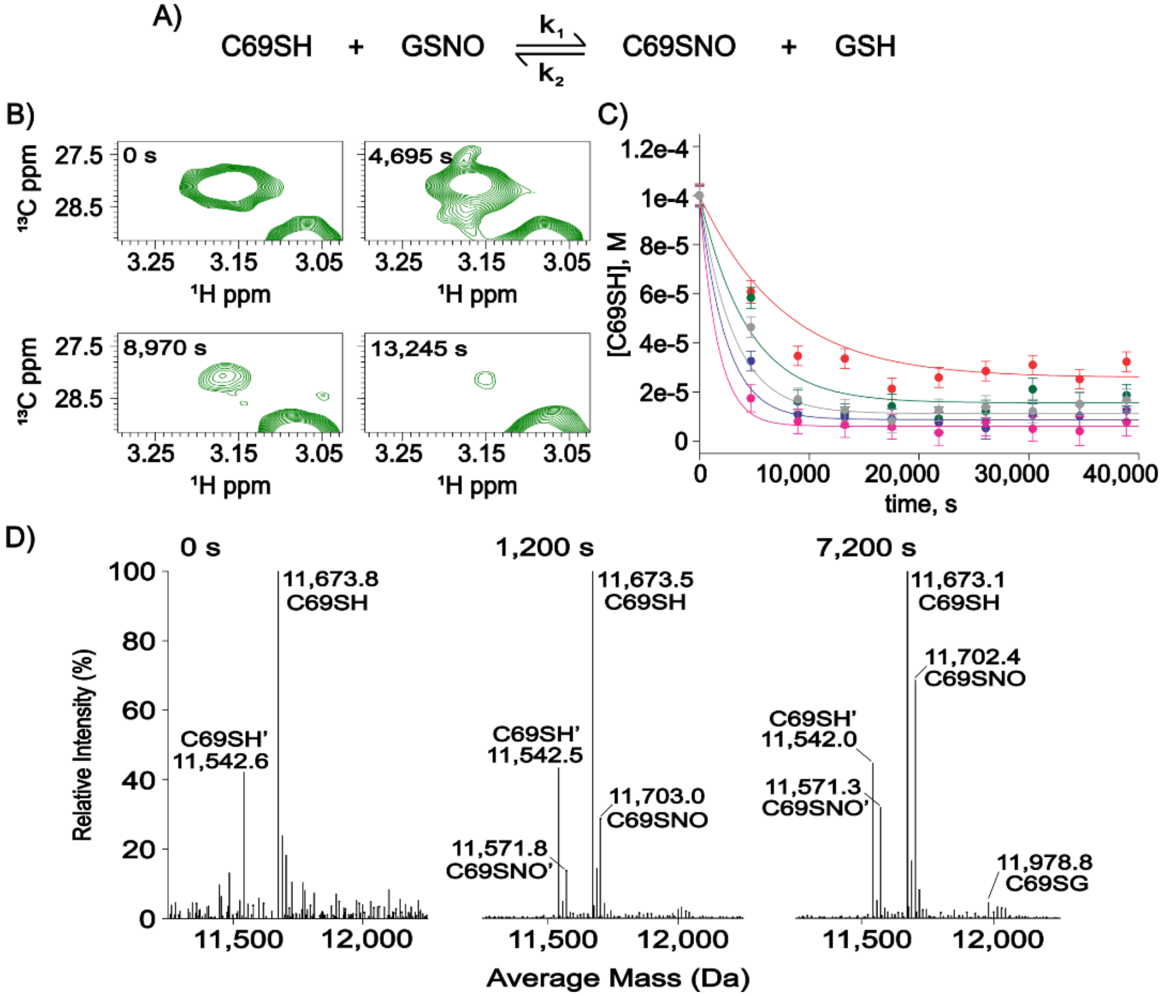

Figure S1: S-nitrosation Kinetic of Cys69only (C69SH). A) The reaction between C69SH mutant and GSNO. **B)** ^{1}H-^{13}C HSQC at different reaction times for reaction between C69SH (100 µM) and 10x of GSNO. It is possible to observe the signal intensity of H_β/C_β of C69SH decreasing over time. **C)** Global fitting of kinetic data calculated based on H_β/C_β of C69SH signal intensity. The curves correspond to different reaction conditions: 5x (red), 10x (green), 15x (gray), 20x (blue) and 30x of GSNO (magenta). The rate constant calculated for the C69SH S-nitrosation reaction was 1.9 ± 0.1 x10^{-1}. **D)** Mass spectra of the reaction between C69SH (100 µM) and 10x of GSNO. We acquired three mass spectra: before adding GSNO (0 s) and after adding it after the 1,200 s and 7,200 s reaction times. At 0 s, only non-nitrosated proteins are observed with (C69SH) and without (C69SH') the initial methionine; At 1,200 s, the presence of nitrosated species C69SNO and C69SNO' has already been verified. At 7,200 s, in addition to the species previously seen, we also detected the formation of S-glutathionylated protein (C69SG). The experimental error for each experimental point in C was estimated from the standard deviation of the spectral noise for one experiment.

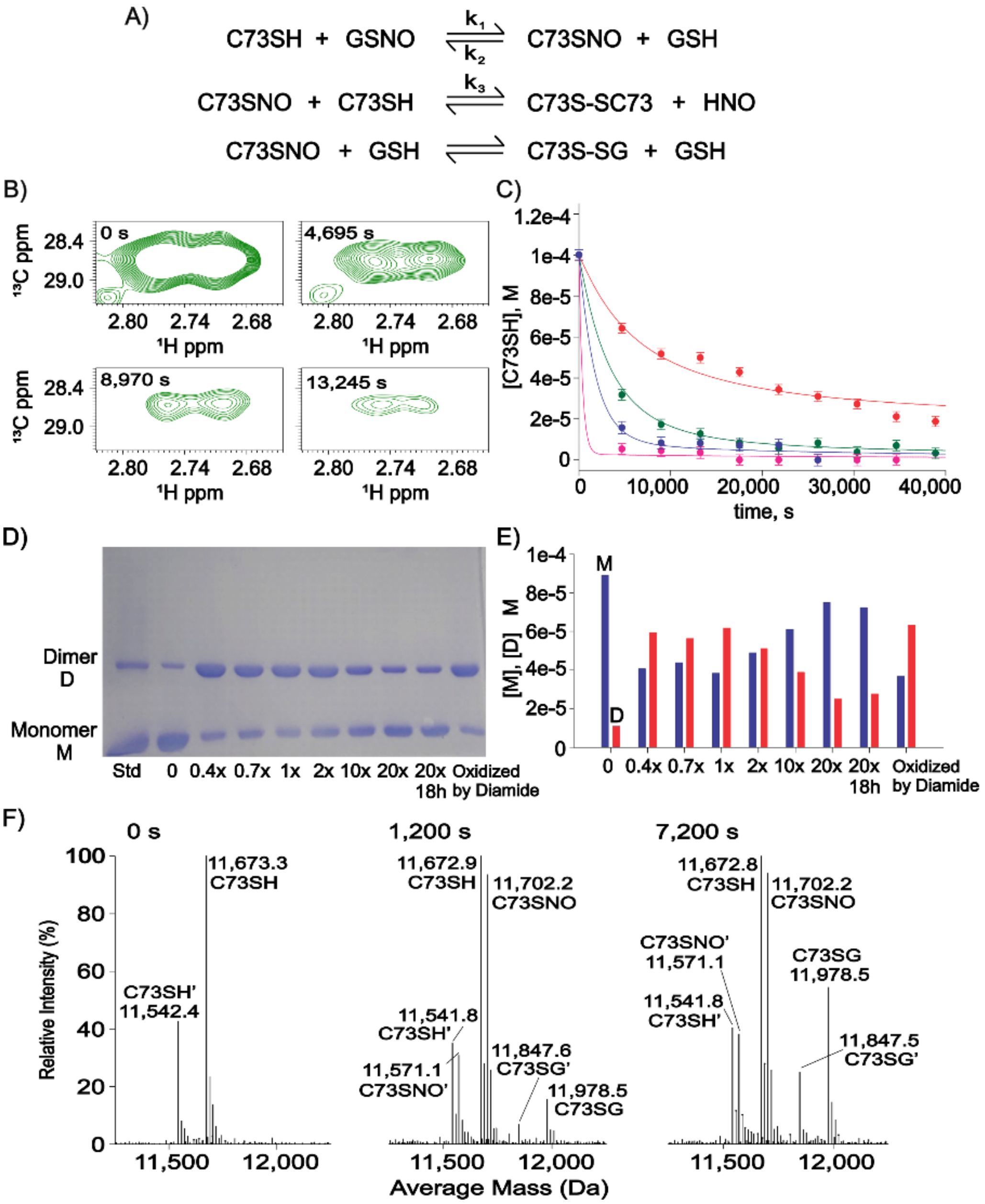

Figure S2: S-nitrosation Kinetic of Cys73only (C73SH). A) The reaction between C73SH mutant and GSNO (first reaction) and consecutive reactions of dimerization e glutathionylation. **B)** ^{1}H-^{13}C HSQC at different reaction times for reaction between C73SH and GSNO, both at 100 µM. It is possible to observe only H_β /C_β cross-peak of the reduced Cys residue and its decrease intensity over time. **C)** Global fitting of the C73only mutant S-nitrosation kinetics by GSNO. The red, green, blue, and magenta curves correspond to the kinetics with 0.5x, 1x, 2x, and 10x of GSNO, respectively. In all curves, it is possible to observe the decay over time in the concentration of C73SH, the non-nitrosated species. By global fitting, it was possible to calculate the k_1 for second-order reaction, whose value was equal to $2.50 \pm 4.6 \times 10^{-1}$ $M^{-1}s^{-1}$. **D)** By SDS-PAGE it was possible to monitor the intensity difference between monomer (C73SH and C73S-SG) and dimer (C73S-SC73) bands insofar as varying quantities of GSNO (0x, 0,4x, 0,7x, 1x, 2x, 10x e 20x) were applied in the kinetic experiments. The gel also shows the result of the experiment with 20x of GSNO after 2h and 18h of reaction, in addition to the oxidation experiment with diamide. In all experiments, the time of reaction was 2 hours. The standard (Std) corresponds to a sample of the C69only mutant with a small dimeric fraction to reference the dimer band. **E)** In the graph, it is possible to observe the variation between monomer and dimer as the concentration of GSNO varies in the medium. **F)** S-nitrosation kinetic of C73only by Mass spectrometry. The three spectra acquired in different reaction times show the species C73SH and C73SH' at 0 s, C73SH, C73SH', C73SNO, and C73SNO' at 1,200 s. After 7,200 s, beyond the species detected at 0 s (without GSNO) and 1,200 s, the mass spectrum shows the species C73SG e C73SG', which correspond to the post-translational modification of S-glutathionylation. The experimental error for

each experimental point in C was estimated from the standard deviation of the spectral noise for one experiment.

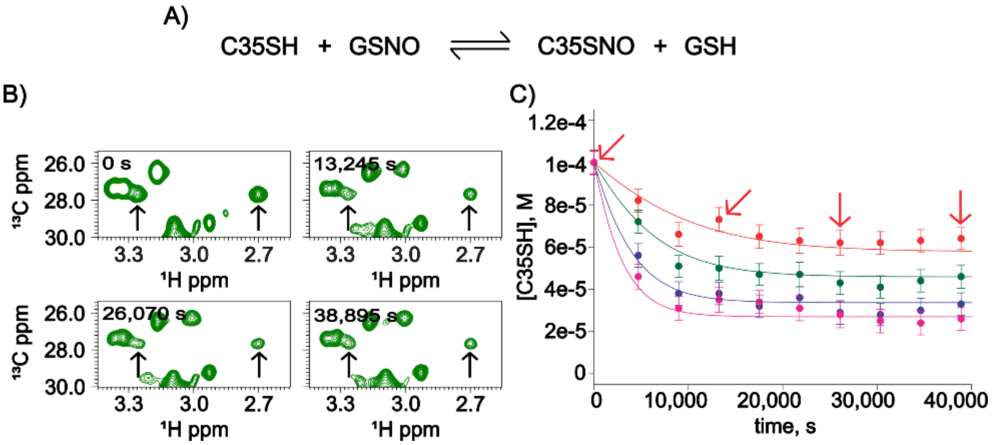

Figure S3: S-nitrosation kinetics of the C35only mutant. A) S-nitrosation reaction of the C35only mutant. **B)** ^{1}H-^{13}C HSQCs acquired at different times of the reaction with an excess of 10x GSNO. We indicated H_{β} e/C_{β} cross-peaks of the free Cys residue with arrows and its intensity decrease over time can be observed. **C)** Global fitting of the C35only mutant S-nitrosation kinetics by GSNO. The red, green, blue, and magenta curves correspond to the kinetics with an excess of 5x, 10x, 20x, 30x of GSNO, respectively. In all curves, it is possible to observe the C35SH concentration decay over time, the non-nitrosated species. From the global fitting carried out with the aid of the KinTek Explorer software, it was possible to calculate the k_1 for second-order reaction, whose value was equal to 7.6 ± 0.7 x10^{-2} $M^{-1}s^{-1}$. The experimental error for each experimental point in C was estimated from the standard deviation of the spectral noise for one experiment.

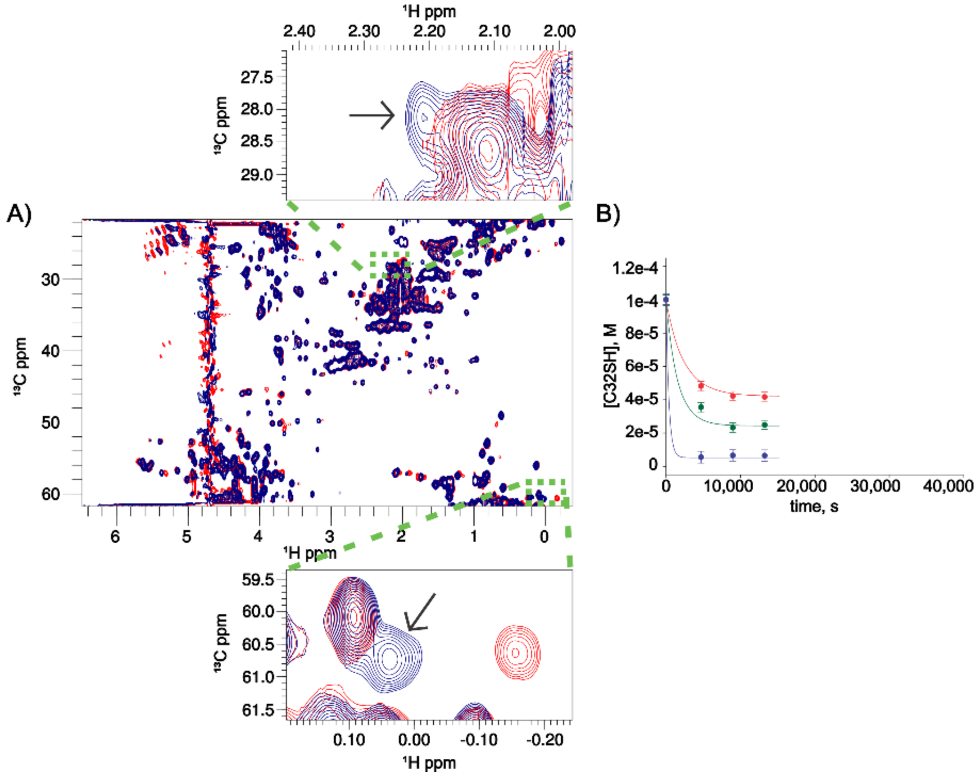

Figure S4: S-nitrosation kinetics of the C32only mutant. A) In the center of the image is the overlap of the ^{1}H-^{13}C HSQCs before (in blue) and after the addition of 10x GSNO (in red). In the upper zoom, an arrow indicates H_{β} /C_{β} cross-peak of residue C32. We found this signal in a region of the ^{1}H-^{13}C HSQC with many signals, so the signal chosen as a probe for the kinetic experiments of this mutant was the signal shown in the lower zoom. **B)** Global fitting of the S-nitrosation kinetics of the mutant C32only by GSNO. The red,

green, and blue curves correspond to the kinetics with 1x, 2x, and 10x of GSNO, respectively. In all curves, it is possible to observe the C32SH concentration decrease over time. From the global fitting carried out with the aid of the KinTek Explorer software, it was possible to calculate the k_1 for second-order reaction that was 2.6 ± 0.3 $M^{-1}s^{-1}$. The experimental error for each experimental point in B was estimated from the standard deviation of the spectral noise for one experiment.

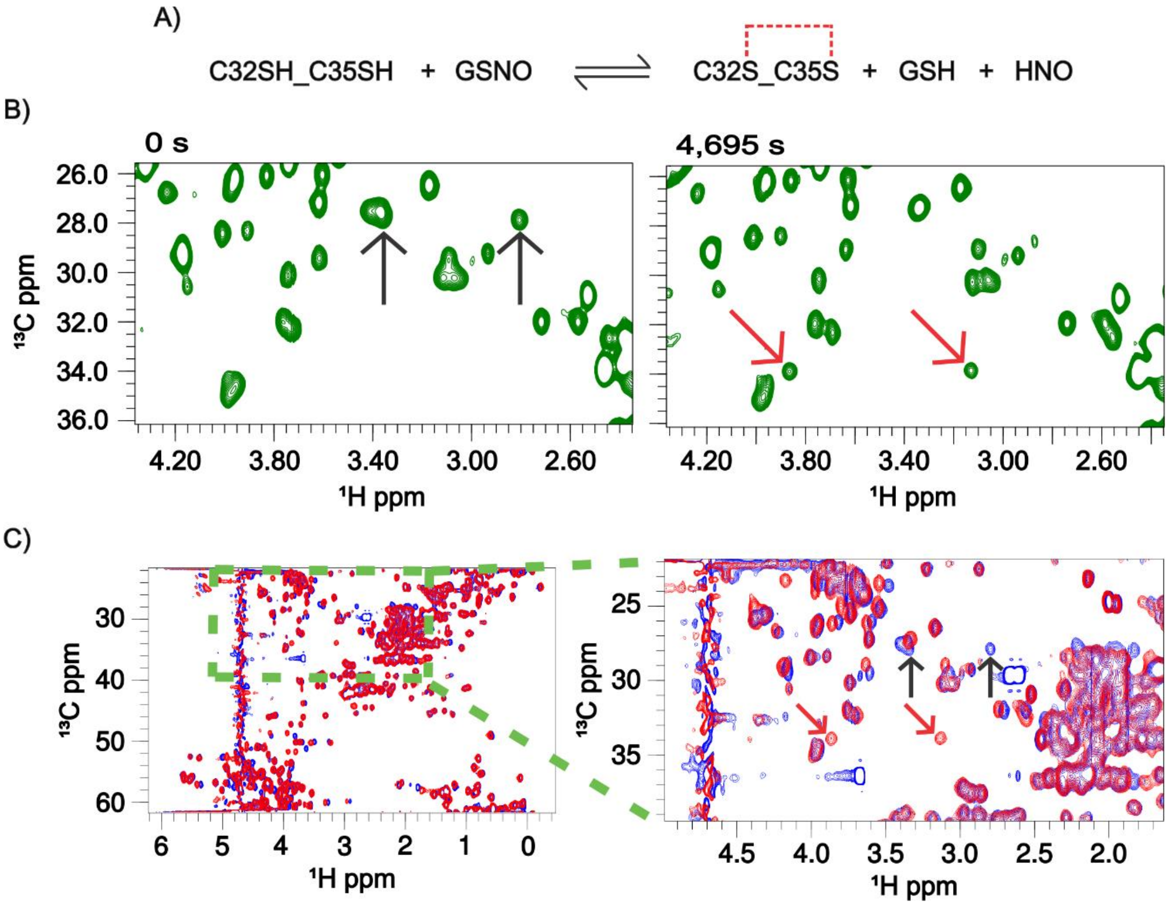

Figure S5: S-nitrosation kinetics of mutant C32C35. A) The reaction between the C32C35 mutant and GSNO leads to oxidation of the redox site. **B)** Zoom of the ^{1}H-^{13}C HSQCs acquired before (0 s) and after (4,695 s) the addition of 10x of GSNO. In the left-hand spectrum, it is possible to observe the H_β /C_β cross-peaks of the non-nitrosated residue C35 indicated by arrows. In the 4,695 s second reaction time, the expansion on the right, the same signs no longer observed. However, signs of oxidation are observed and indicated by red arrows. **C)** Overlapping of ^{1}H-^{13}C HSQCs of mutant C32/C35. In blue, the ^{1}H-^{13}C HSQC of the C32/C35 mutant acquired in the presence of the reducing agent DTT and, in red, ^{1}H-^{13}C HSQC of the C32/C35 mutant oxidized with diamide. The zoom shows reduced H_β /C_β cross-peaks of C35 residue indicated by black arrows and the oxidized signs, by red arrows.

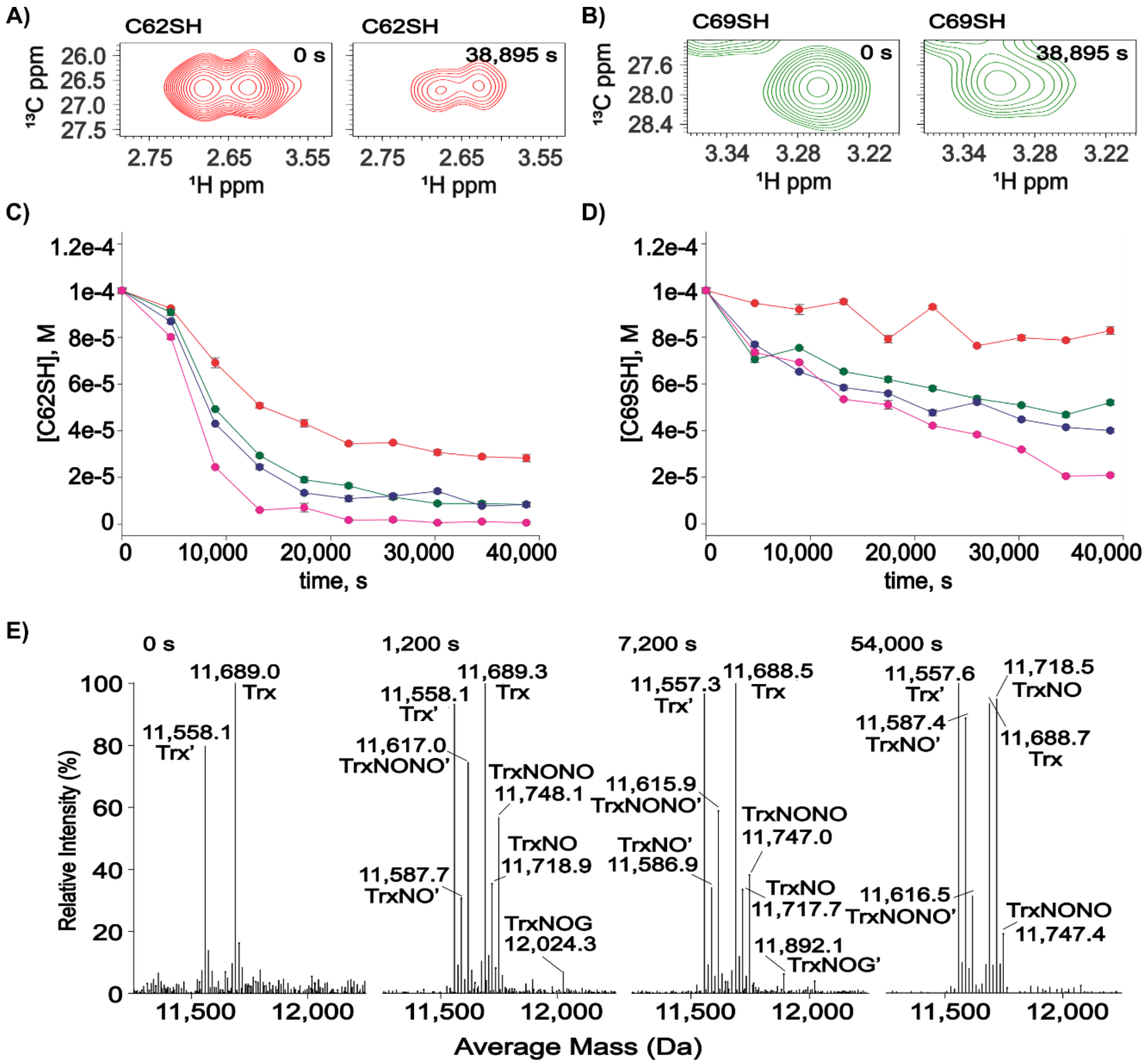

Figure S6: S-nitrosation kinetic of C62C69 mutant. A) ^{1}H-^{13}C HSQCs acquired at different times of the reaction with an excess of 10x GSNO. The figure shows the non-nitrosated H_β/C_β cross-peak C62 intensity decrease over time. **B)** ^{1}H-^{13}C HSQCs expansions showing just the C69 residue signal. **C)** C62SH and D) C69SH concentrations decreasing over time. The red, green, blue, and magenta curves correspond to the kinetics with an excess of 10x, 20x, 30x, and 40x of GSNO, respectively. **E)** S-nitrosation kinetics of C62C69 mutant by mass spectrometry. Four mass spectra were acquired for the kinetics with an excess of 10x of GSNO, namely: before adding GSNO, 0 s, and after adding it after the 1,200 s, 7,200 s, and 54,000 s reaction times. On the spectra, for the sake of clarity, the C62C69 mutant with the initial methionine is represented by Trx, without methionine by Trx'. At 0 s, only non-nitrosated proteins Trx and Trx' are observed. At 1,200 s, it was already possible to verify, in addition to the presence of the once nitrosated species (TrxNO and TrxNO'), the double nitrosated species TrxNONO and TrxNONO', and the species once nitrosated and glutathione TrxNOG. We observed such species on the 7,200 s and 54,000 s spectra. The experimental error for each experimental point in C and D was estimated from the standard deviation of the spectral noise for one experiment.

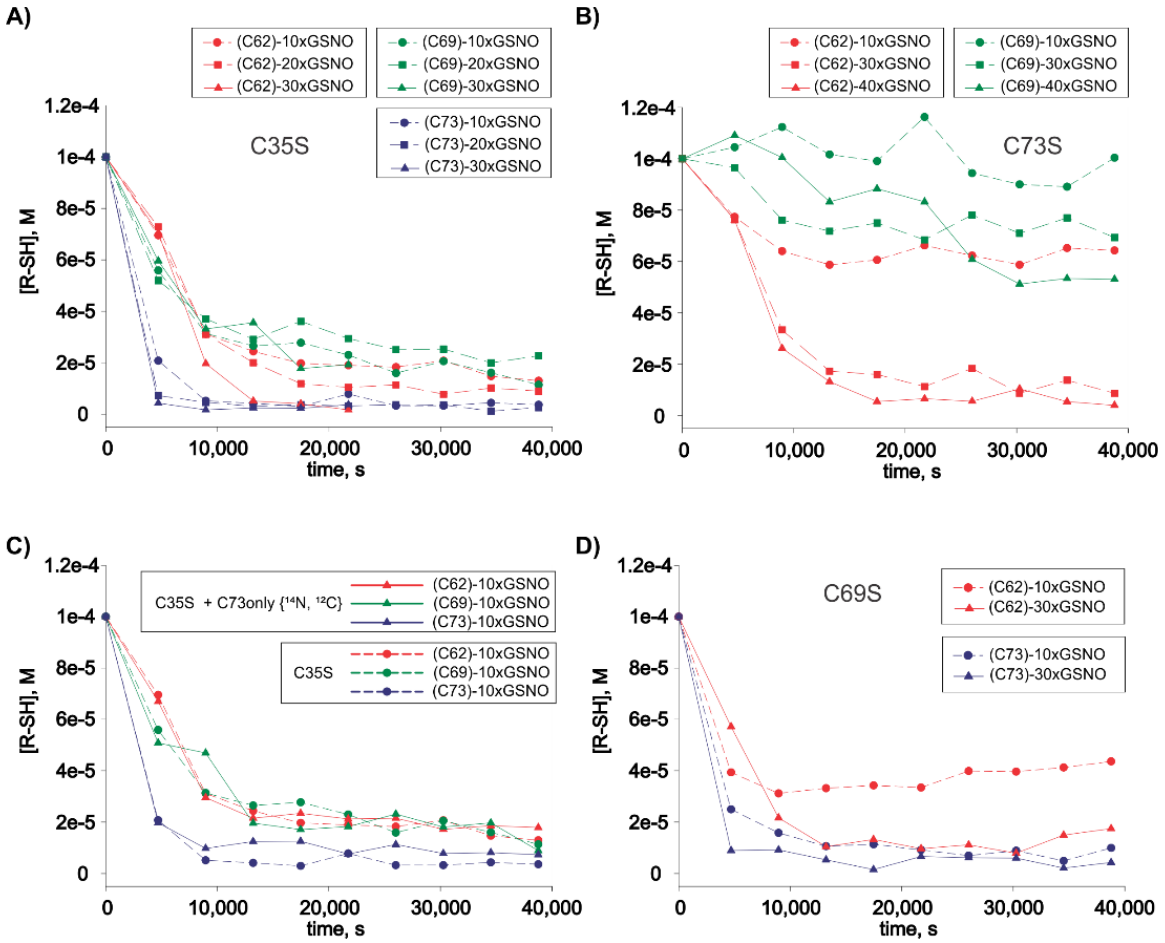

Figure S7: S-nitrosation kinetics of mutants with multiple S-nitrosation sites. A) S-nitrosation kinetics of the C35S mutant. The graph shows C62SH, C69SH, and C73SH concentration decay over time. The red, green, and blue curves correspond to the S-nitrosation kinetics of C62, C69, and C73 residues, respectively, with an excess of 10x, 20x, and 30x of GSNO. For each kinetics, the concentration of each non-nitrosated cysteine residue was monitored over time. The graph shows that at the beginning of the kinetics, the second point of each curve, the C62SH concentration remained higher than the C69SH concentrations, which showed a slightly greater decay initially. C73 is the most reactive residue, as it showed the greatest decrease. **B)** S-nitrosation kinetics of the C73S mutant. The graph shows the C62SH and C69SH concentration decay over time, which we represented generically by R-SH in the graph. The red and green curves correspond to the nitrosation kinetics of cysteines C62 and C69, respectively, with an excess of 10x, 30x, and 40x GSNO. For each kinetics, we monitored the concentration of each non-nitrosated cysteine residue over time. **C)** S-nitrosation kinetics of the C35S mutant in the presence of C73only. The graph shows the decay of the concentration of C62SH, C69SH, and C73SH for the S-nitrosation kinetics experiment of mutant C35S in the presence of 100 µM of mutant C73only and an excess of 10x of GSNO. The solid lines represent the curves for this experiment. The colors red, green, and blue correspond to the measures for residues C62, C69, and C73, respectively. The graph also shows the results obtained in the S-nitrosation kinetics of the C35S mutant with 10x GSNO, which are represented by dashed lines with the same color pattern for each residue. Comparing the curves from the two experiments (C35S S-nitrosation with and without C73only), it is not possible to observe relevant changes in the decay profile of the residue concentration curves. This suggests that the C73 residue does not provide an intermolecular NO transfer mechanism. **D)** S-nitrosation kinetics of the C69S mutant. The plot shows the C73SH concentration decay in blue, and C62SH, in red. To each Cys residue, we observe two curves derived from kinetics experiments with 10x and 30x of GSNO. C73SH decay is faster than C62SH one, and the profiles of each residue are similar to that observed in wild type hTrx. The experimental error for each experimental point was estimated from the standard deviation of the spectral noise for one experiment.

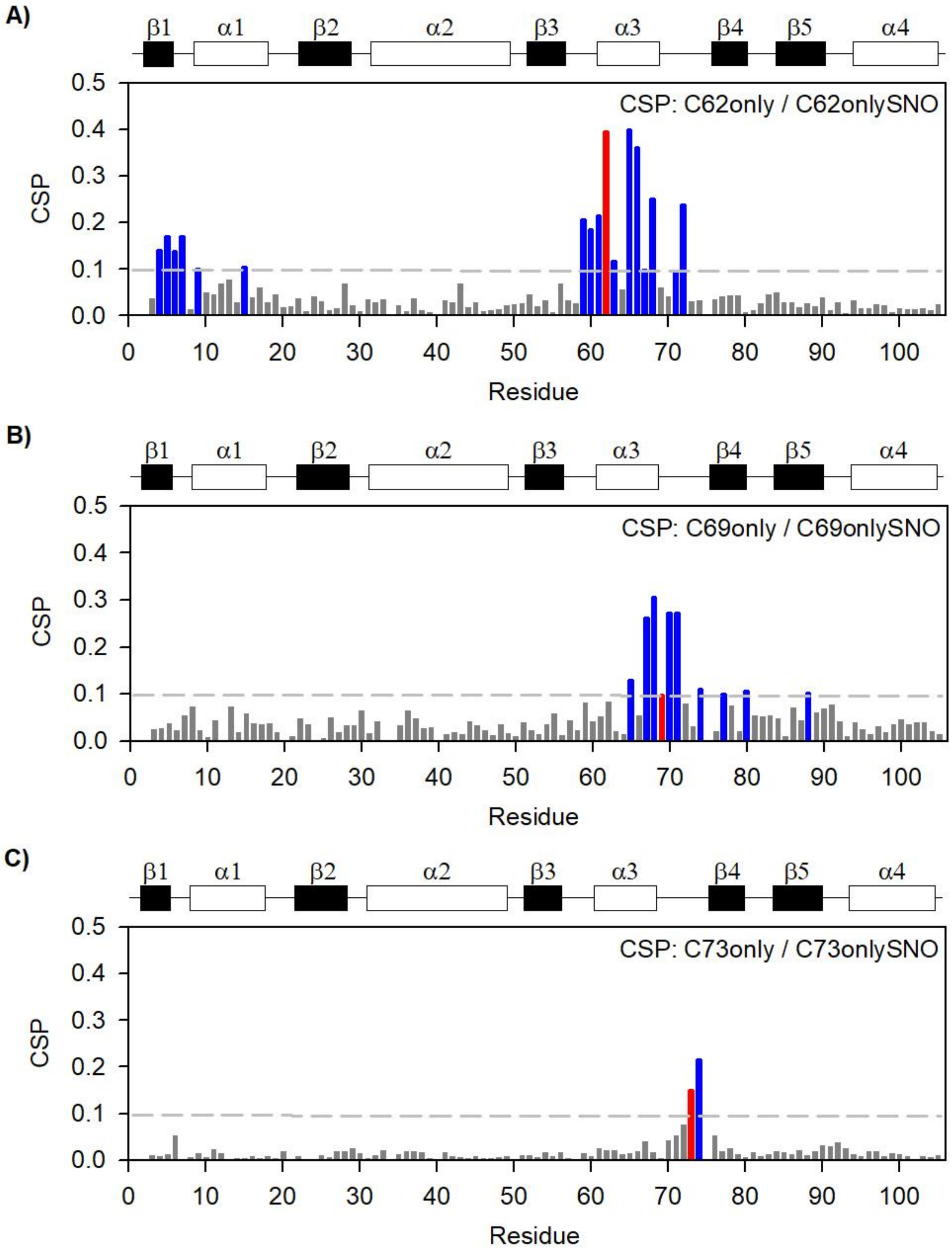

Figure S8: ^{1}H-^{15}N Chemical Shift Perturbation (^{1}H-^{15}N CSP). The red bar represents the S-nitrorasion site, the blue bar represents all residues that CSP was over 0.1 and the gray bars are residues that CSP was below 0.1. Above each plot, black and white boxes indicate β-sheet and α-helix respectively. **A)** S-nitrosation of C62only mutant resulted in remarkable CSP at β1, β1-α1 loop, and close regions to C62 residue, β3-α3 loop, α3 and α3-β4 loop. **B)** S-nitrosation of C69only mutant elicited CSP over 0.1 in residues mainly at α3 and α3-β4 loop. **C)** S-nitrosation of C73only mutant resulted in noteworthy CSP just at C73 and M74 residues, probably due to the great solvent-exposed of C73 residue.

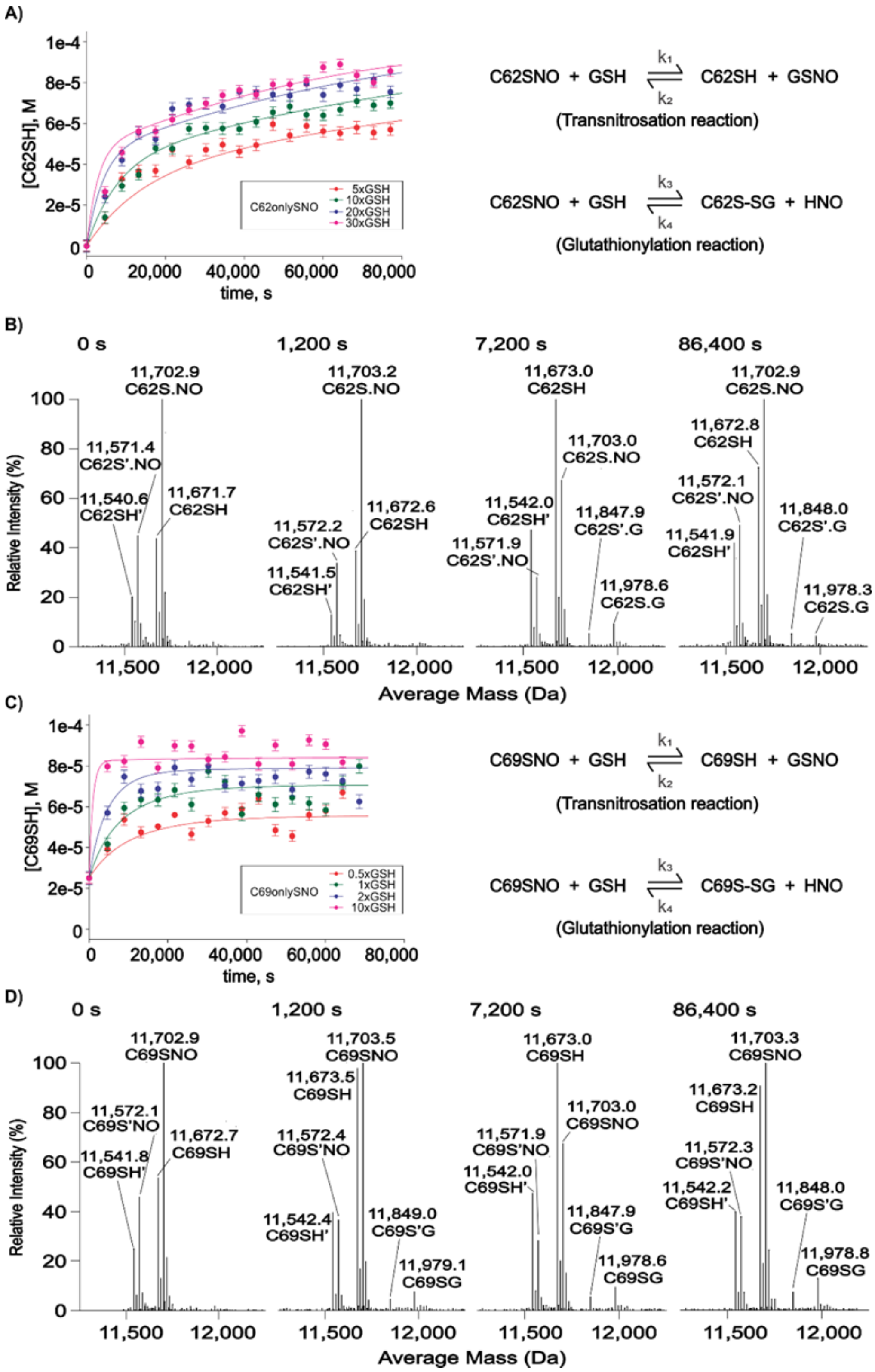

Figure S9: Transnitrosation reaction from C62only(SNO)/C69only(SNO) to GSH. A) Global fitting of transnitrosation reaction between C62only(SNO) mutant and GSH at different concentrations by NMR. Besides the transnitrosation reaction (k_1 = 5.9 ± 0.6 x10^{-2} M^{-1} s^{-1}), we considered the S-glutathionylation reaction (k_3 = 4.9 ± 1.2 x10^{-2} $M^{-1}s^{-1}$). **B)** Mass spectrometry data of reaction between 100 μM of protein and 1 mM of GSH confirmed the transnitrosation as well as the S-glutathionylation reaction. **C)** We also globally fit the transnitrosation reaction (k_1 = 8.4 ± 1.2 x10^{-1} M^{-1} s^{-1}) data between C69only(SNO) mutant and GSH. Note that to this reaction less amount of GSH was necessary and S-glutathionylation reaction (k_3 = 2.3 ± 1.0 x10^{-1} $M^{-1}s^{-1}$) was also considered. **D)** Mass spectrometry data of C69onlySNO and GSH reaction.

The experimental error for each experimental point in A and C was estimated from the standard deviation of the spectral noise for one experiment.

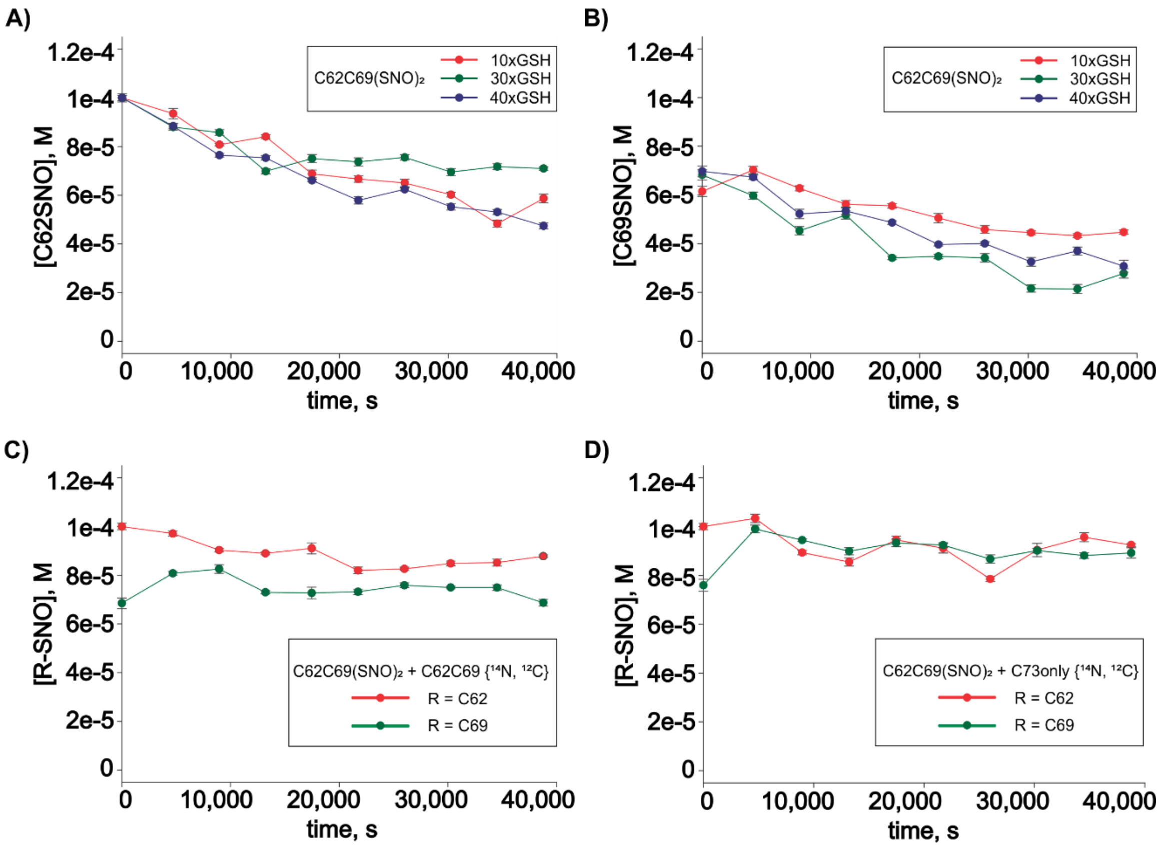

Figure S10: Transnitrosation reaction of C62/C69(SNO)$_2$ to different targets. Note that all transnitrosation reactions of C62C69(SNO)$_2$ were very slow. **A)** Decrease of C62SNO concentration due to transnitrosation reaction to GSH. **B)** Decrease of C69SNO concentration due to transnitrosation reaction to GSH. **C)** Transnitrosation reaction between C62/C69(SNO)$_2$ $\{^{15}N, ^{13}C\}$-labeled and C62/C69 $\{^{14}N, ^{12}C\}$ mutants. Red dots represent C62SNO concentration over time and green dots, C69SNO one. **D)** Transnitrosation reaction between C62/C69(SNO)$_2$ $\{^{15}N, ^{13}C\}$-labeled and C73only $\{^{14}N, ^{12}C\}$ mutants. Red dots represent C62SNO concentration over time and green dots, C69SNO one. The experimental error for each experimental point was estimated from the standard deviation of the spectral noise for one experiment.
